# Improving ergonomics in the operating room for orthopaedic surgeons in order to reduce work-related musculoskeletal injuries

**DOI:** 10.1016/j.amsu.2020.06.020

**Published:** 2020-06-23

**Authors:** Motaz Alaqeel, Michael Tanzer

**Affiliations:** Division of Orthopaedic Surgery, McGill University, Montreal, Quebec, Canada

**Keywords:** Ergonomics, Injury, Orthopaedic, Operating room, Work, Musculoskeletal

## Abstract

**Introduction:**

Orthopaedic surgery is characterized by surgical tasks that are physical, repetitive and require some degree of stamina from the surgeon. Occupational injuries are alarmingly common in orthopaedic surgery with two-thirds of all surgeons reporting a work-related musculoskeletal (MSK) injury during their career. One of the leading causes of the high level of MSK injuries among orthopaedic surgeon is lack of ergonomics of the operating room. Implementing an ergonomic process has been shown to be effective in reducing the risk of developing MSK disorders in other high-risk industries. We reviewed well-established and effective ergonomic guidelines from the industrial workplace and determined the pertinent principles that could be transferred to the operating room to help reduce the number and severity of common orthopaedic work-related MSK injuries.

**Methods:**

We reviewed the ergonomic guidelines, primarily from the Occupational Safety and Health Administration (OSHA), that specifically address minimizing the risk of these work-related injuries and that are transferable to the operating room. In addition, the ergonomic guidelines from the Canadian Center for Occupational Health and Safety (CCOHS), the National Institute for Occupational Safety and Health (NIOSH) and the Centers for Disease Control and Prevention (CDC) were reviewed.

**Results:**

Many of the guidelines to avoid work-related injuries in industry are transferable to the operating room. The pertinent guidelines clearly indicated how to adjust the height of the operating table, the proper design of hand and power tools and the modifications to the operating room environment that can help prevent injury. These guidelines from industry include maintaining a neutral posture and joint alignment, working with the appropriate hand tools and minimizing the lower extremity fatigue by using the proper footwear and floor mats.

**Discussion:**

Optimizing the occupational environment and utilizing well-established ergonomic principle from industry is both feasible and practical in the operating room to decrease the incidence of musculoskeletal injuries among this high-risk profession. These guidelines are simple, effective and are easy to implement by orthopaedic surgeons in order to minimize their risk of sustaining a work-related injury.

## Highlights

• Work-related injuries in Orthopaedic Surgery are common.
• Industry has developed various guidelines to avoid work-related injuries.
• Many of these guidelines are simple, effective and transferable to the operating room.
• Proper instruments, power tools and posture are effective strategies.

## 1 Introduction

Combining the direct and indirect costs, occupational injuries are estimated to cost more than 190 billion annually in the U.S, due to lost productivity [1]. The Occupational Safety and Health Administration (OSHA) has defined an injury or illness as work-related if an event or exposure in the work environment either caused or contributed to the resulting condition, or significantly aggravated a pre-existing condition [2]. Orthopaedic surgery is generally a physically demanding surgical specialty that predisposes orthopaedic surgeons to sustain a variety of work-related musculoskeletal (MSK) injuries [[3], [4], [5]]. This is likely due to both the physical nature of the surgery, as well as the poor ergonomics of the work environment [6,7]. Work-related physical injury to the surgeon can result in time away from work, thereby having a temporary financial and psychological effect on the practicing surgeon, or at times, a permanent effect on the surgeon's career.

In the context of the operating room, where repetitive/sustained awkward postures, high task repetition and forceful exertions commonly seen, ergonomics is particularly important to reduce the risk of injury [8]. The word ergonomics comes from the Greek words “ergon,” meaning work, and “nomos,” meaning natural laws or arrangements. It is the study of people at work and how the working environment is designed to suit the worker [9]. To date, there is a shortage of ergonomic studies conducted in the operating room [10]. Most of the literature related to improving ergonomics to minimize MSK injury has been related to video endoscopic surgery [9,[11], [12], [13]]. The inefficient layout of the operating room and the maintenance of awkward static body postures present some of the largest ergonomics challenges [10]. The conditions that surgeons withstand have been compared with those of certain industrial workers and therefore, it not surprising that some ergonomic principles from non-medical industries has provided some helpful guidelines for surgeons in the operating room [12]. These include the importance of correct body posture to decrease strain on the neck and lower back, patient positioning and table height [9].

Occupational injuries are alarmingly common in orthopaedic surgery with two-thirds of all surgeons reporting a work-related MSK injury during their career, of which 27%–31% required time off work ranging from a half day to forced retirement [[3], [4], [5]]. The most common reported MSK injuries involve the neck and lower back, followed by upper extremity injuries, including shoulder and rotator cuff disease, lateral epicondylitis tendinitis, and carpal tunnel syndrome. One of the leading causes of the high level of MSK injuries among orthopaedic surgeon is the lack of ergonomics of the operating room. Implementing an ergonomic process has been shown to be effective in reducing the risk of developing MSK disorders in other high-risk industries [6]. Despite the availability of multiple studies regarding the ergonomic environment in the operating room, there is a lack of clear and concise guidelines.

In this article, we reviewed well-established and effective ergonomic guidelines from the industrial workplace and determined the pertinent principles that could be transferred to the operating room to help reduce the number and severity of common orthopaedic work-related MSK injuries. Specifically, we reviewed the ergonomic guidelines established by the primary government organization responsible for workplace safety and public health, in both the US and Canada, to identify these ergonomic guidelines and standards in the workplace.

## 2 Methods

In the US, the Occupational Safety and Health Administration (OSHA) is the primary governmental organization responsible for safe and healthful working conditions, while the Centers for Disease Control and Prevention (CDC) is the leading national public health institute responsible for protecting public health and safety through the control and prevention of injury and disability [14,15]. The CDC focuses on occupational safety and health through its research agency, the National Institute for Occupational Safety and Health (NIOSH) [16]. In Canada, the Canadian Center for Occupational Health and Safety (CCOHS) is the primary federal government corporation that is mandated to promote workplace health and safety [17].

We reviewed OSHA's ergonomic guidelines, that specifically address minimizing the risk of work-related injuries in workers, similar to orthopaedic surgeons, from prolonged standing, repetitive work and/or heavy manual work [6,18]. In addition, the ergonomic guidelines from the CCOHS, NIOSH and the CDC were reviewed [[19], [20], [21], [22]]. All the industry guidelines that related to the most common MSK injuries sustained by orthopaedic surgeons, and could be transf erable to the operating room environment are discussed [3].

## 3 Results

The federal organizations provided effective ergonomic guidelines from the industrial workplace that specifically addressed the most common reported MSK injuries, including the lower back and neck; the upper extremity injuries, including shoulder and rotator cuff disease, lateral epicondylitis tendinitis, and carpal tunnel syndrome; and the lower extremities and feet.

According to OSHA, the workspace should be designed so that workers are performing approximately 15% or less of their maximum capacity [23]. Therefore, to minimize fatigue and to reduce the amount of effort exerted on the muscles, the surgeon should work while maintaining a neutral joint posture for as much of the surgical procedure as possible. Operating rooms and surgical instruments should be designed to enable surgeons to work as close as possible to a neutral posture.

There are established guidelines in the manufacturing, construction and agricultural industries that have been developed to decrease back injury among their employees. Some of these recommendations are pertinent to surgeons and can be adopted by orthopaedic surgeons in the operating room to diminish the risk of back and neck injuries. According to CCOHS, table height plays a significant role in decreasing the strain on the back muscles, minimizes neck flexion and minimizes leaning and reaching over [20]. The table height should also vary based on the nature of task performed. Performing a precise task such as soft tissue dissection or mobilizing critical structures requires the table height to be about 5 cm above the level of your elbow (elbow height). For light work such as screw insertion or suturing, the table height should be approximately 5–10 cm below the elbow height. However, when performing heavy tasks, and downward forces are needed, such as drilling or impacting a component with a mallet, the table height is recommended to be 20–40 cm below the elbow height [24]. There are several simple measures advocated by OSHA to address the frequent need to lean forward or reach during surgery. Firstly, the patient should be placed as close as possible to the surgeon on the operating room bed. Secondly, tilting the bed toward the surgeon will maximize the operative field visualization and minimize both the need to lean and bend over the patient [23].

Ideally while operating, the surgeon's arm should maintain a neutral posture in order to minimize fatigue and injury to the upper extremities [25]. According to NIOSH, the neutral posture of the shoulder is 0° of adduction without flexion, extension or shoulder abduction [25] **(**Fig. 2). The elbow position varies according to the type of task needed to be accomplished [25]. For the majority of surgical tasks, maintaining the elbow joint closer to 90° of flexion provides functionality and avoids fatigue, especially when performing tasks for an extended period of time [25] (Fig. 3). In addition, according to CCOHS, for tasks requiring precision the elbow joint can be flexed up to 110°, and when performing a heavy task the elbow should be in approximately 40–50° of flexion [19]. Ideally, NIOSH recommends the neutral posture of the wrist joint should be maintained in order to decrease the stress that is applied to the joint while working as prolonged, or excessive palmar flexion of the wrist is associated with carpal tunnel syndrome [25,26] (Fig. 4).

**Fig. 1:**
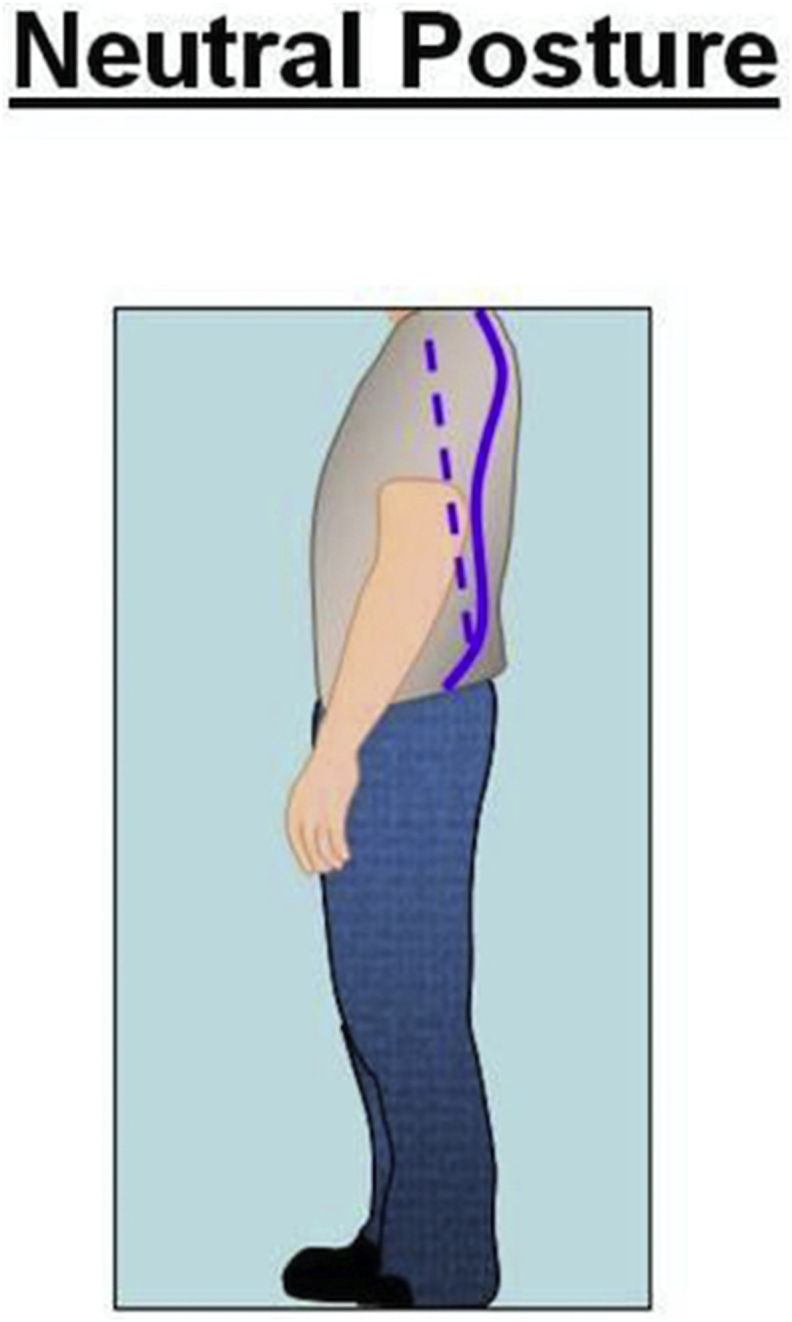
Illustration of the neutral posture for the back and the neck during standing. In the sagittal plane is when that the ears are aligned over the shoulder, and the shoulder over the hips and the hips over the knees, and knees over the ankles. The spine should maintain a neutral (S) shape curvature, with cervical spine lordosis and slight thoracolumbar kyphosis and lumbar lordosis.

**Fig. 2:**
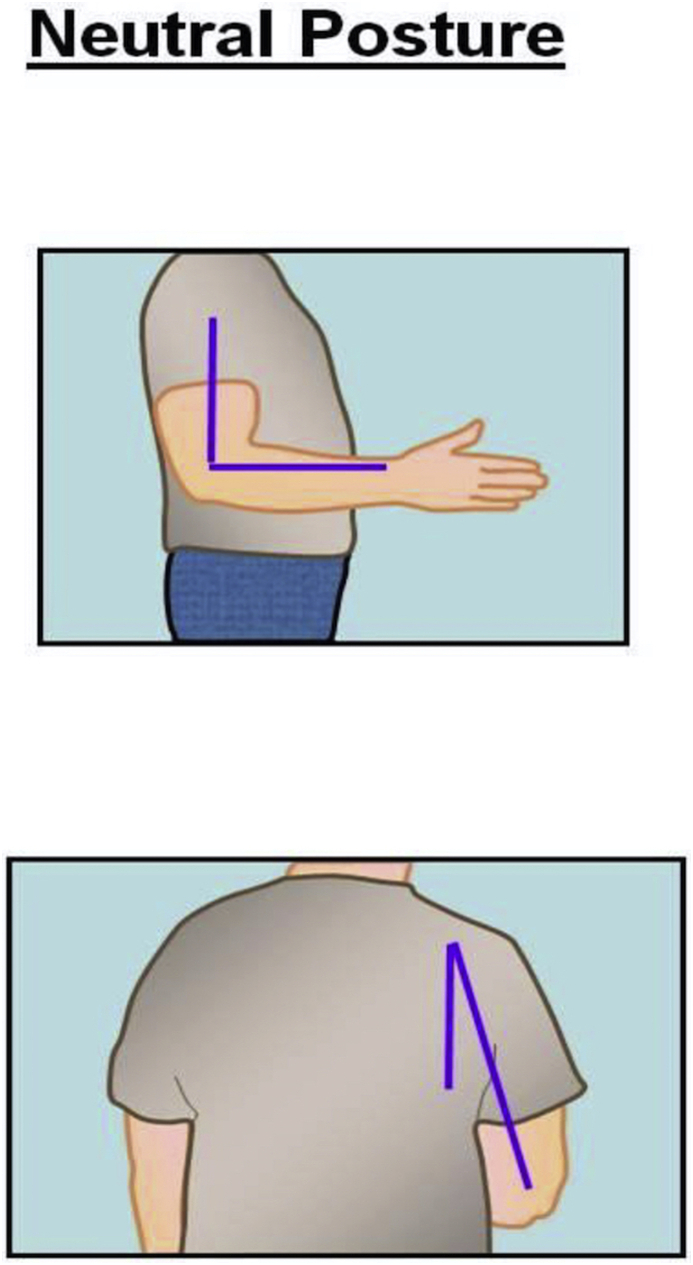
Illustration of the neutral positions, on coronal and sagittal plains, for the shoulder in standing position.

**Fig. 3:**
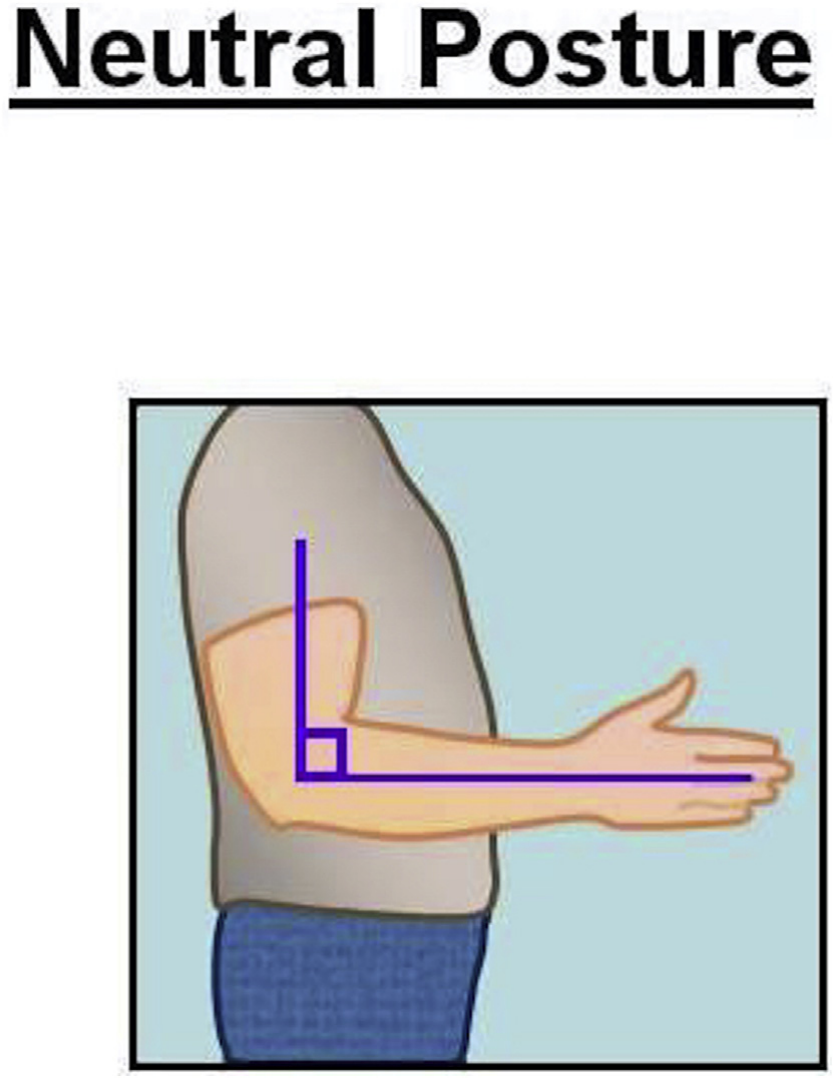
Illustration of the neutral position on sagittal plains for the elbow.

**Fig. 4:**
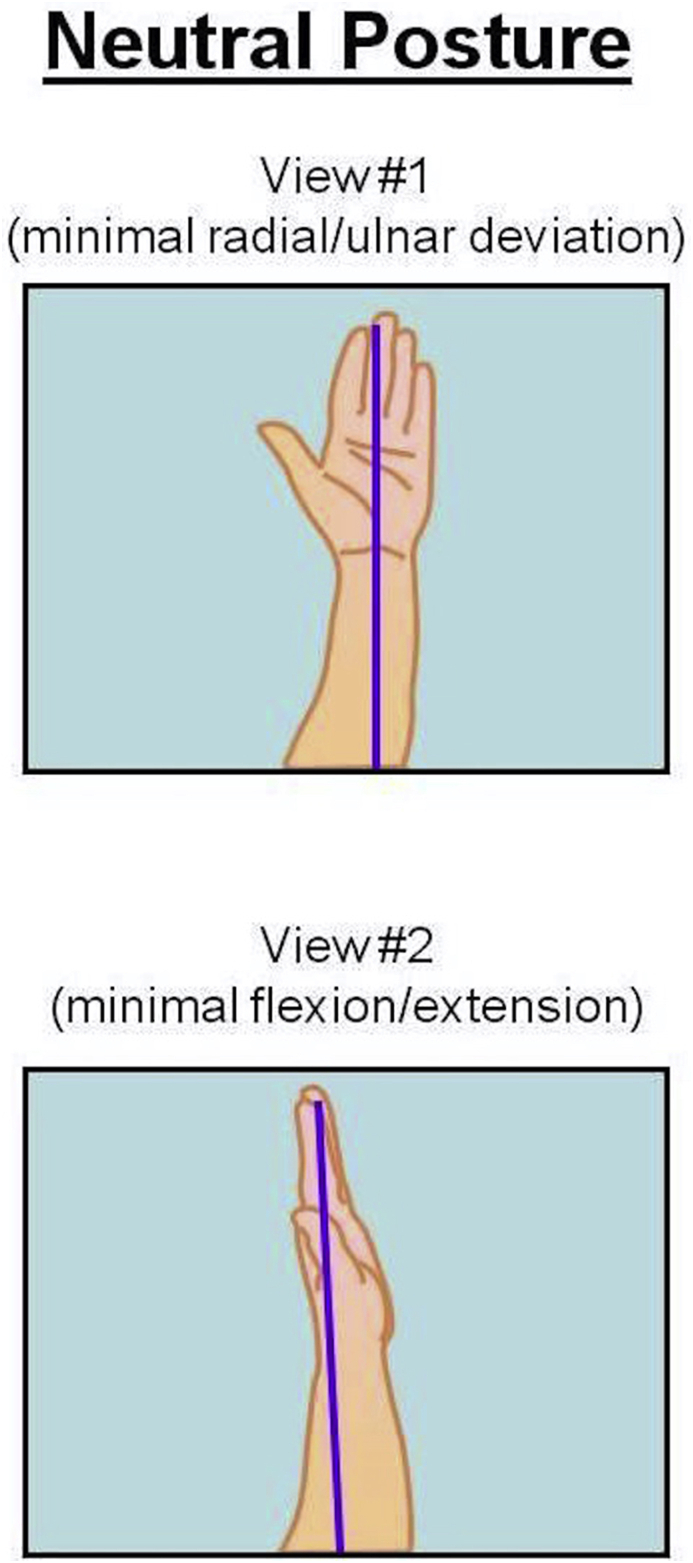
Illustration of the neutral position of the wrist joint in the sagittal and coronal plains. The neutral position of the wrist is that position where the wrist is in straight alignment with the forearm, with no flexion, extension, radial or ulnar deviation, and at the mid-point between supination and protonation.

**Fig. 5:**
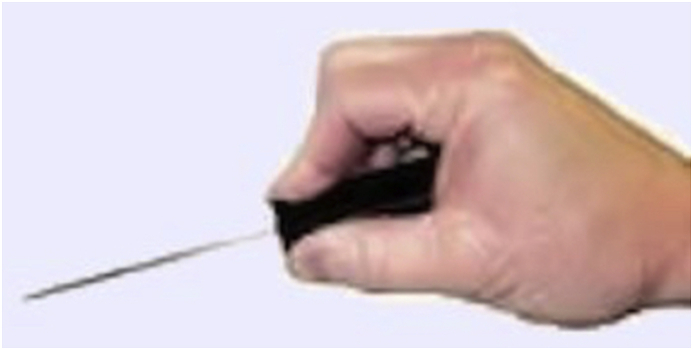
Illustration of the pinch grip: The tool is gripped between the thumb and the fingertips.

OSHA and NIOSH have developed a consensus document which provided guidelines to select the proper ergonomically designed hand tools in order to minimize the MSK injuries in the workplace [22]. These guidelines are relevant and transferable to orthopaedic surgery, where hand tools are frequently used (Table 1). When a task requires precision and accuracy, a pinch grip is used, and the tool is gripped between the thumb and the fingertips [20] (Fig. 5). To optimize ergonomics, the diameter of the handle or grip span will then depend on whether the tool is being used for a power or a precision task. For single handle tools intended to perform a heavy task with a power grip, like a mallet, the handle diameter of the tool should be between 1.25 and 2 inches. When precision is needed, a single handle tool is held with a pinch grip with handle diameter between 0.25 and 0.5 inches [20]. Doubled handle tools used for precision, like forceps or Metzenbaum scissors, should have a grip span with a minimum width of 1 inch when fully closed, and no more than 3 inches when fully open. When an object must be held for a prolonged time with a constant force, the use of a clamp or locking pliers is recommended [21]. Double handle tools used for power tasks, such as pliers, should have an open grip span of no more than 3.5 inches, and a closed grip span not less than 2 inches [22]. In order to prevent contact pressure between the end of the handle and the palm, the length of the handle should be longer than the widest part of the hand, usually 4–6 inches [22]. The grip surface for the handle should be slightly compressible and smooth [22,27]. The compressible material decreases vibration and allows better distribution of pressure. When metallic handles are used, its recommended to encase then with a plastic sheet [22]. There should not be finger grooves on the handle [22]. Maintaining a neutral wrist alignment is important when using hand tools and can be best achieved using different handle offsets [22]. When a force is needed to be applied horizontally, an offset handle is recommended. However, when the force is needed to be applied vertically, a straight handle provides a better wrist alignment in order to generate a higher force [21]. A mallet or hammer is widely used in the orthopaedic surgery and misusing it can result in injury [28]. According to CCOHS, the mallet should be held by the end of the handle [28]. To reduce fatigue, the hammer should weight not more than 2.3 kg, with an ideal weight between 0.9 and 1.75 kg [29]. To increase efficiency and provide optimum energy transfer, the striking surface should be parallel to the surface being struck. To generate more power, the swing should start from the shoulder and elbow, with the wrist in neutral position [28]. However, wrist motion is preferred when fine control and precision is required [28,30].

**Table 1:** Summary of consensus guidelines for the selection of ergonomically designed hand tools.

Descriptions	Guideline	Reason
**Handle Shape**	Cylindrical or slightly contoured	Easy and control grip
**Horizontal Direction Of Force**	Offset handle	Minimal wrist deviation
**Vertical Direction Of Force**	Straight handle	Minimal wrist deviation
**Single Handle Diameter (Power Grip)**	1.25–2 inch	Greater force and stability
**Single Handle Diameter (Pinch Grip)**	0.3–0.6 inch	Greater control
**Doubled Handle Diameter (Power Grip)**	Closed: > 2 inch	Maximum grip strength
	Opened: <3.5 in	
**Doubled Handle Diameter (Pinch Grip)**	Closed: > 1 inch	Maximum grip strength
	Opened: <3 inch	
**Handle Length**	>4 inch	Avoid palm contact pressure

**Fig. 6:**
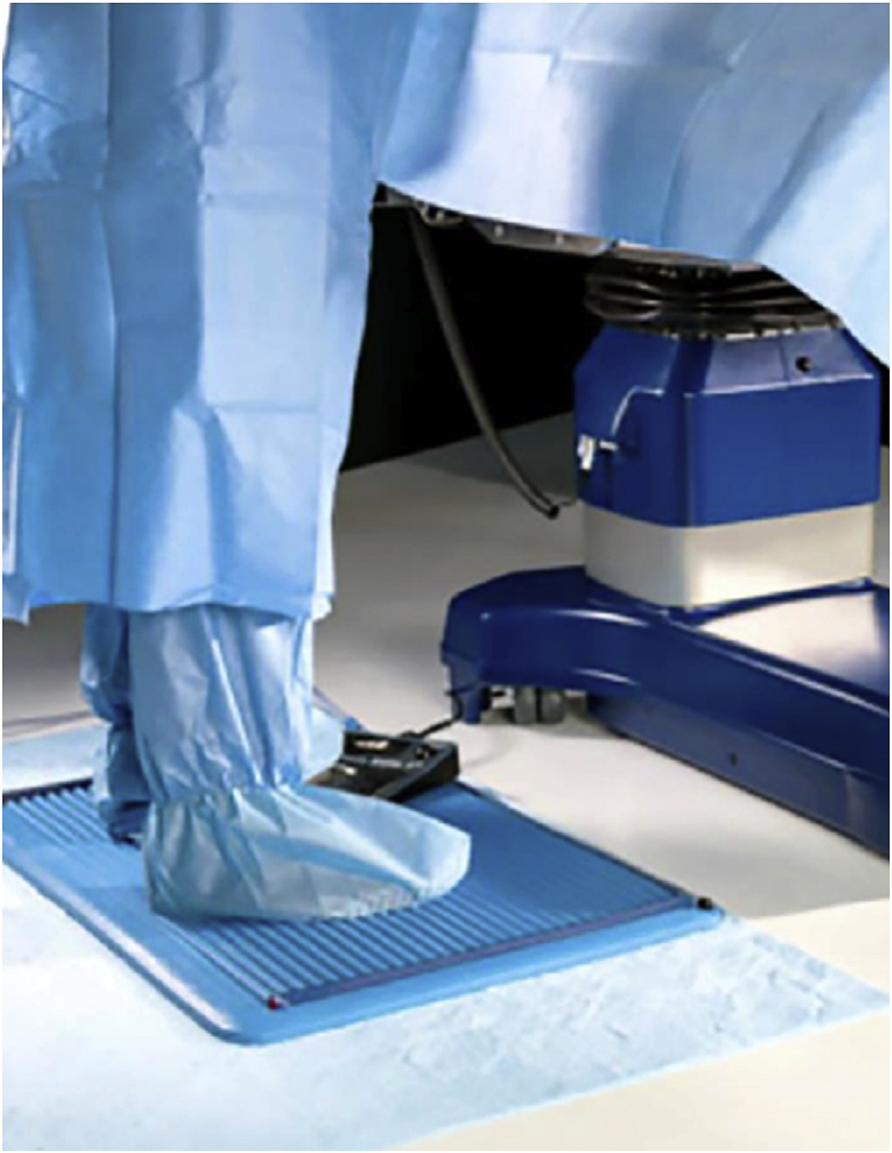
Photograph of an anti-fatigue mat.

OSHA and the CCOHS recognize that standing with a static posture for a prolonged period of time subjects the workers to fatigue and possible injuries [[31], [32], [33]]. In many industries, like construction, manufacturing or assembly line work, there are guidelines to reduce lower extremity complaints, such as sore feet, lower limb edema, general muscular fatigue, and low back pain that are that are transferable to the operating room for orthopaedic surgeons [19,30]. CCOHS has identified footwear as playing an important role in preventing lower extremity complaints [34]. Flat shoes should be avoided as they increase the strain on the Achilles tendon. CCOHS recommends to use shoes with a heel between 1 and 2.5 cm [19]. As well, shoes should allow toe freedom, and provide arch support [24,34]. Both OSHA and CCOHS recommend the use of anti-fatigue mats to minimize foot discomfort when standing for an extended period of time on a hard surface[[19], [25], [32], [35]]. These fatigue reducing mats are made of various materials, such as rubber or vinyl, and function by absorbing and distributing the pressure of standing on the lower extremities and back, thereby reducing foot fatigue (Fig. 6). However, caution is required, as the use of matting can lead to tripping and falling accidents [19]. In addition, CCOHS recommends the use of a footrest to allow the shifting of one's weight from one foot to the other, in order to relieve the strain on the lower back [34,36]. Since workers that stand prefer to adopt an asymmetrical standing position over a symmetrical one, a foot rest can have a significant benefit in tasks that require prolonged standing [36]. In addition, standing with one leg on a footrest reduces the intervertebral disc stress by preventing excessive lumbar spine lordosis [36]. Ideally, CCOHS advises the footrest height should be approximately 15–20 cm and in 20–30° of inclination [24].

## 4 Discussion

Government organizations have been created to ensure safe working conditions by setting and enforcing standards, and by providing education to avoid work-related injuries [14]. Many of these standards have to do with the appropriate ergonomics in the workplace. Many industries have successfully implemented these ergonomic solutions in their facilities to successfully reduce the number of MSK injuries [37]. Although these industry guidelines do not specifically target surgeons, they contain several recommendations and best practices that are transferable to the operating room. The guidelines outlined in this study may assist orthopaedic surgeons in minimizing the well-established work-related musculoskeletal (MSK) injuries that are common in their profession, and are in part due to the poor ergonomics of their work environment [[3], [4], [5], [6], [7]]. These guidelines from industry specifically address ergonomic strategies to minimize back, arm, and lower extremity injuries of orthopaedic surgeons in the operating room by optimizing OR equipment; surgical posture; the use and design of power and hand tools; and shoe wear.

While industrial ergonomics is well recognized and has made a significant impact on their workers, penetration into the domain of surgery is only now improving, notably in Minimally Invasive Surgery [9,[11], [12], [13]]. However, similar recognition and improvements in ergonomics in the operating room for orthopaedic surgeons, whose workload is harsher than other surgical subspecialties, is lacking. Furthermore, many surgeons are not aware that some guidelines do exist to help prevent these prevalent MSK occupational injuries [38]. In industrial ergonomics it is well recognized that neutral posture is essential to minimize the stress applied to muscles and tendons, thereby decreasing the risk of MSK injury. Awkward posture is a known risk factor for MSK injury and should be avoided during surgery [7]. Surgeons can generate the highest amount of force when the joint in a neutral posture. When joints are aligned toward the extreme of the ranges, the muscles and tendon are either elongated or contracted, which results in a loss of maximum control and force generation [39,40]. As a result, the surgeon has to work harder and expend more energy compared to that in a neutral posture. This makes the surgeon prone to fatigue and a MSK injury. In addition, orthopaedic surgical procedures can be long in duration and are commonly performed without rest or interruption. Therefore, preserving energy and avoiding fatigue is critical to avoid a MSK injury [40]. The spine is particularly vulnerable and more than 60% of orthopaedic surgeons sustain a work-related injury to their spine [6]. This is likely related to open surgical procedures that almost always require standing, awkward body positions, with frequent and prolonged static head-bent and back-bent postures, instead of the spine being in a neutral position [[41], [42], [43], [44]] (Fig. 1). Several studies have demonstrated a high prevalence of upper extremity injury among orthopaedic surgeon, with almost 50% of orthopaedic surgeons sustaining an upper extremity injury during their surgical career [3,45]. The most common injuries reported are lateral epicondylitis of the elbow (18%) and shoulder tendonitis (14%), with rates similar to that reported among worker that perform repetitive manual tasks, such as assembly line and packaging workers [46]. Therefore, it seems reasonable to extrapolate the strategies to prevent injury from these industries to the operating room.

There are several limitations to this study. Firstly, although the ergonomic guidelines outlined have been successful in industry, this study did not evaluate these guidelines in the operating room to determine how successful they would be in orthopaedic surgery. The guidelines in this study were highly selected from the primary government organization responsible for workplace safety and public health, in both the US and Canada, and did not include other organization in North America or elsewhere. Although other organizations may have important ergonomic guidelines for the workplace, the organizations reviewed are the government organizations responsible for all workers in North America. In addition, NIOSH has an international understanding of occupational safety since it accomplishes its mission in partnership both nationally and international groups. Although the organizations making these guidelines may not have evaluated all industries, many of the guidelines are universal, and we specifically looked at those that were related to well-established orthopaedic MSK injuries related to the work environment. Finally, the study does not take into account or test other ergonomic strategies that are not identified by these governmental organizations, that may be equally, or more effective in the operating room.

Orthopaedic surgery is almost exclusively focused on the patient, while the surgeon is neglected and thereby prone to work-related MSK injuries. Despite all the advances in surgical technology over the past few decades, the operating room is still not ergonomically designed. In addition, orthopaedic tools are still primarily designed with the working end in mind, with little attention to the importance of the surgeon's end, and its potential for causing injury. Industry provides an opportunity to utilize well-established and successful ergonomic principles to minimize the risk of MSK injury in orthopaedic surgeons. However, for this to be a recognized standard of safety, there is a need to make orthopaedic surgeons aware of these ergonomic issues and educate them on the relevant industry guidelines [38].

## 5 Conclusion

Orthopaedic surgery is a physically demanding profession, with a high prevalence of musculoskeletal injury [3]. Optimizing the occupational environment and utilizing well-established ergonomic principle from industry is both feasible and practical in the operating room to decrease the incidence of musculoskeletal injuries among this high-risk profession. Maintaining a neutral alignment, working with the appropriate hand tools, minimizing lower extremity fatigue by using the proper footwear and floor condition may help in preventing some of these injuries. These guidelines are simple, effective and are easy to implement by orthopaedic surgeons in order to minimize their risk of sustaining a work-related injury.

## Consent

No patients in the study. Not required.

## Annals of medicine and surgery

The following information is required for submission. Please note that failure to respond to these questions/statements will mean your submission will be returned. If you have nothing to declare in any of these categories then this should be stated.

## Ethical approval

Research studies involving patients require ethical approval. Please state whether approval has been given, name the relevant ethics committee and the state the reference number for their judgement.

None required.

## Funding

None.

## Author contribution

Michael Tanzer - Concept, design, data analysis and interpretation, editing. Motaz Alaqueel - Data collection, data analysis, writing, image copyright approvals.

## Registration of research studies

In accordance with the Declaration of Helsinki 2013, all research involving human participants has to be registered in a publicly accessible database. Please enter the name of the registry and the unique identifying number (UIN) of your study.

You can register any type of research at http://www.researchregistry.com to obtain your UIN if you have not already registered. This is mandatory for human studies only. Trials and certain observational research can also be registered elsewhere such as: ClinicalTrials.gov or ISRCTN or numerous other registries.

1 Name of the registry: Not required. No patients involved in the study
2 Unique Identifying number or registration ID:
3 Hyperlink to your specific registration (must be publicly accessible and will be checked):

## Guarantor

Dr Michael Tanzer.

## Provenance and peer review

Not commissioned, externally peer reviewed.

## CRediT authorship contribution statement

**Motaz Alaqeel:** Data curation, Formal analysis. **Michael Tanzer:** Conceptualization, Formal analysis, Writing - review & editing.

## Declaration of competing interest

None.
